# Interprofessional collaboration associated with frequency of life-saving links to HIV continuum of care services in the urban environment of Newark, New Jersey

**DOI:** 10.1186/s12913-020-05866-3

**Published:** 2020-11-07

**Authors:** Liliane Cambraia Windsor, Rogério Meireles Pinto, Carol Ann Lee

**Affiliations:** 1 University of Illinois at Urbana-Champaign, 1010 W. Nevada Street, Urbana, IL 61801 USA; 2 University of Michigan, Ann Arbor, USA

**Keywords:** Substance-use disorder (SUD) treatment, Interprofessional collaboration, HIV testing, HIV continuum of care

## Abstract

**Background:**

HIV continuum of care has been used as a strategy to reduce HIV transmission rates, with timely engagement in HIV testing being the first and most critical step. This study examines interprofessional-collaboration (IPC) after controlling for agency/ provider demographics, provider training and self-efficacy as a significant predictor of how frequently HIV service providers link their clients to HIV testing.

**Methods:**

Multilevel binary logistic regression analysis was conducted to examine the effects of IPC on links to HIV testing while controlling for demographic and agency information, provider training, and standardized measures of providers’ feelings, attitudes, and opinions about IPC. Cross-sectional data from 142 providers in 13 agencies offering treatment and prevention services for HIV and substance-use disorders were collected via a survey.

**Results:**

Those who scored higher on the IPC scale reported significantly higher rates of linkages to HIV testing. Compared to the null model (i.e., no predictor model), the final multilevel binary logistic regression model showed a significantly improved likelihood of linkage to HIV testing by 11.4%, p. < .05. The final model correctly classified 90.2% of links to HIV testing. Providers in agencies with smaller budgets and in agencies offering substance use disorder services were more likely to link clients to HIV testing. Younger providers who received HIV training were also more likely to link clients to HIV testing.

**Conclusions:**

Findings suggest IPC training as a potential strategy to improve linkages to HIV testing for clients at risk for HIV infection. Future research is recommended to identify specific areas of IPC that might have differential effects on links to HIV testing.

**Supplementary Information:**

The online version contains supplementary material available at 10.1186/s12913-020-05866-3.

## Background

While much progress has been made reducing the transmission of the human immunodeficiency virus (HIV) globally, significant health inequalities among underserved populations remain. In the United States (US), Black people accounted for 13% of the US population and 42% of the 37,832 new HIV diagnoses in 2018 [1]. Newark, the largest city in the state of New Jersey, is an example of an American city that is struggling to recover from the loss of manufacturing jobs since the 1980s. Newark’s population is predominantly comprised of Black people, with a median annual household income of $33,139 [2]. Newark has the largest number of people living with HIV in New Jersey, with 1404 out of every 100,000 residents living with HIV, compared to 299.5 nationally [1]. Nearly 70% of these individuals are Black people even though Black people constitute 53% of the Newark population [3]. Moreover, Newark has the highest number of people with substance-use disorders (SUD) in the state [4]; with as many as 30% injecting drugs intravenously. These individuals are at higher risk for acquiring HIV, for being underserved, and are more likely to be diagnosed at a late stage of HIV infection [5, 6].

In the past decade, great progress in decreasing the number of people with acquired immunodeficiency syndrome (AIDS) has been achieved by increasing coverage of antiretroviral therapies (ART) in people living with HIV. The World Health Organization recommends that individuals at risk be tested for HIV; initiate ART as soon as possible after diagnosis [7]. ART lowers the viral load in the bloodstream, making transmission of HIV less likely to occur [8]. Research has shown that individuals living with HIV who engage in ART consistently show an undetectable viral load for at least 6 months, have a negligible risk of transmitting the virus to other people [9]. Taken together, HIV testing, linkage to care, and viral suppression through ART constitute the HIV continuum of care (“care continuum”), recommended since 2012 as a preventive measure to reduce rates of HIV transmission worldwide [9]. Research shows that in the US, only 55% of individuals living with HIV adhere to treatment and 28 to 35% achieve viral suppression [10]. An estimate of the impact of treatment and prevention on HIV incidence in Newark shows that increased treatment adherence and HIV testing were the most effective interventions to reduce HIV incidence [11].

Early detection of HIV infection with subsequent ART treatment are widely documented as cost-saving and effective in extending life expectancy, enhancing life quality, and reducing HIV transmission [12]. Therefore, the most critical element of early HIV treatment is timely engagement with HIV testing, the first step in the care continuum [13]. UNAIDS established “90–90-90” treatment goals in 2015, whereby 90% of all people infected with HIV will be diagnosed, 90% of those diagnosed with HIV will receive ART, and 90% of those on ART will achieve undetectable HIV viral-load suppression by 2020 [14]. However, attaining the 90–90-90 goal among individuals with high HIV exposure risk will require more-rigorous efforts to find, test, and link individuals to HIV primary care treatment.

Individuals more likely to be exposed to HIV include those who experience comorbidities, such as untreated mental disorders and substance misuse, and those less likely to be engaged in care [15]. People of color experience disproportionately higher HIV-care dropout rates and reduced levels of viral suppression. These findings are exacerbated by high undiagnosed rates among these underserved populations. For example, a recent study indicated that only 6.7% of study participants with severe mental illness were tested for HIV over a yearlong period [16], and another study reported that most homeless people do not have access to HIV testing [17]. Furthermore, Whites had a lower percentage undiagnosed compared with other ethnic groups [18]. Thus, it is critical to identify socio-medical factors that may contribute to these inequalities while identifying factors that can help reduce them [19, 20].

### The role of providers in the continuum of care

The care continuum engages those living with HIV (both diagnosed and undiagnosed) in a sequence of evidence-based services—HIV testing, primary HIV care, and ART—provided by medical professionals. HIV testing, the first step, holds particular importance because all other steps are contingent upon the results of such testing. Most individuals access HIV testing through members of a diverse workforce of providers of social and public health services (e.g., social workers, health educators, care navigators), who, in their day-to-day practices, link individuals to medical personnel who can offer HIV testing and other subsequent services. A socio-medical approach to understanding engagement in the care continuum reveals that factors at the provider level (e.g., work experience), the interpersonal level (e.g., positive attitudes toward interprofessional collaboration), and the environmental level (e.g., best HIV-prevention practices) exert influences on how and how often providers engage individuals in the care continuum by linking them to HIV testing, HIV care, and other support services [21].

Research has shown that integrating HIV testing with SUD treatment is an effective and feasible strategy to identify undiagnosed individuals living with HIV suffering from SUD [22–24]. Yet only 28% of SUD-treatment programs offer HIV testing on site [25]. Providers and administrators report that the difficulties of obtaining organizational support and finding continuing funding are the most significant barriers to integration of HIV-testing and SUD-treatment services [22]. Thus, linking clients to HIV testing, treatment, and prevention may be a useful strategy for SUD-treatment programs that do not offer onsite HIV services. In the U.S., HIV prevention has historically consisted of behavioral counseling, offered by social and public health providers, which incorporates evidence-based strategies such as sexual-risk assessment, risk-reduction counseling, male and female condom instructional demonstrations, and referrals to HIV testing [26]. More recently, pre/post exposure prophylaxis and treatment as prevention have shifted the focus to linking individuals to care [27, 28]. Increased provider knowledge and on-the-job training in these strategies have been shown to reinforce the practice of linking clients to HIV testing [29].

The literature shows that provider and agency factors may affect referral to HIV testing and care, which ultimately affect the rates of viral suppression and HIV transmission. Interprofessional collaboration (IPC)—in which providers with different job titles and roles work together to help the same clients—has been shown to improve links to services across healthcare systems [30–32]. Literature examining the impact of IPC in HIV-service link-making is scant, particularly in SUD-treatment programs that incorporate HIV prevention as an important part of their services. Research shows that treatment for SUD ought to include substantial HIV-risk reduction [33], a priority recommended by federal and state agencies [34, 35]. However, providers need to be knowledgeable about HIV transmission, symptoms of HIV, and preventive behaviors (e.g., male and female condom use) to do so.

### Conceptual approach

This study employs a socioecological perspective, including four domains of reference: community (agency); relationships (interprofessional); individual (provider); and policy (best practices) [36]. The community domain includes structural and functional factors of agencies, including the services offered and the agency size. The relationships domain includes interpersonal characteristics and capacities (self-efficacy and training about evidence-based HIV-prevention interventions). The individual domain includes factors regarding provider knowledge bases and demographic characteristics. A recent study found that HIV-prevention training was associated with service providers’ increased performance of sexual-risk assessments, risk-reduction counseling, condom demonstration, and referrals for HIV testing [37]. The policy domain reflects the idea that all the other domains are influenced by guidelines provided by funders and health-related organizations (e.g., the World Health Organization and the Centers for Disease Control and Prevention). Specifically, the current study examines whether IPC is a significant predictor of referrals for HIV testing among SUD-treatment and HIV-prevention service providers in Newark, NJ, after controlling for the effects of demographics, provider training, attitudes, and self-efficacy.

## Methods

### Study overview

We followed principles and practices of community-based participatory research (CBPR) to conduct this study. CBPR requires that researchers and community members work together to identify community problems and solutions through a combination of scientific and experiential knowledge [38, 39]. CBPR involves community members in all aspects of the research process, from identifying what the problem is to developing the research methodology, recruiting participants for and conducting the study, analyzing the results, and applying the findings. This approach is an action-oriented paradigm of research. The key purpose of CBPR is to gather knowledge that can be used immediately to help the community involved in the research.

The Newark Community Collaborative Board (NCCB) [40] participated in every stage of the research. The board has 20 members: service providers, consumers, local residents, and researchers. Every board member received training in substance-use epidemiology and treatment, HIV prevention, and CBPR principles, practice, and methods. The NCCB works diligently to distribute power equitably, value both scientific and experiential knowledge, and maintain open dialogue and transparency (http://newarkccb.org/). It is modeled on the New York City Implementation Community Collaboration Board (ICCB), whose mission is to conduct ongoing research on HIV/AIDS, incarceration, and SUD in underserved communities [41]. The current study represents an extension of Interprofessional Collaboration for Implementation (Project ICI), a five-year, NIMH-funded study in New York City, designed to examine factors that facilitate or hinder the care continuum (e.g., HIV testing) and support services (e.g., substance-misuse treatment) that help prevent HIV transmission.

### Recruitment, sampling, and data collection

The NCCB reached out to 21 eligible agencies and successfully recruited a purposeful sample of 142 providers in 13 nonprofit agencies throughout Newark that offer treatment and prevention services for SUD and HIV. The Institutional Review Boards at Rutgers: The State University of New Jersey and Columbia University oversaw the study. We obtained a list of agencies providing such services in Newark using google, the Substance Abuse and Mental Health Administration website, and input from the NCCB (*N* = 20). NCCB members contacted the agencies via phone and email to introduce the study and invite them to participate. Thirteen agencies that agreed to participate secured conference rooms where providers who volunteered to participate could be interviewed in privacy. Trained NCCB members conducted the anonymous survey directly on a laptop loaded with password-protected survey software powered by DatStat Illume 6.0. To be eligible, providers had to consent to participate in the study, offer prevention and treatment services for SUD and/or HIV, and/or be in a position to refer clients to services on the care continuum. Each interview lasted approximately 60 min and included questions about provider demographic information, standardized measures of IPC, implementation of evidence-based interventions, and provider training, and the dependent-variable question about links to HIV testing. The interview guide/survey developed for this study is provided as Additional file 1. Each agency received a $500 cash incentive to participate, and each service provider received a $30 incentive to complete the questionnaire.

### Measurements

#### Outcome variable

Links to HIV testing were measured with a single question: “How often did you link clients to HIV testing within the past six months?” (“Have not referred”; “Referred about once per month”; “Referred about once per week”; “Referred several times per week”). This variable was recoded as dichotomous, with “Have not referred” =0; “Referred at least once per month” =1. This allowed us to focus on the differences between the large number of providers in the data who reported not making any referrals in the past 6 months.

#### Control variables

Four sets of variables were used as controls. The first set referred to agency level variables and included: a) organizational annual income, as a proxy for agency size and b) whether the agency provided SUD treatment in addition to HIV-prevention services, as a proxy for agency capacity.

The second set of variables included provider demographics. *Gender, Ethnicity, and race* were dichotomous and coded as: 0 *=* male and 1 = female; 0 = non-Latino and 1 = Latino; 0 = non-White and 1 = White. *Age* was measured in years. *Education* and *income* were ordinal.

The third set of variables included training and self-efficacy. Training measured included single self-report questions, [1] “Do you have formal training (curriculum-based) in HIV prevention?” (0 = “No” and 1 = “Yes”) [2]; “Have you received training to help clients access HIV testing and primary care? (0 = “No” and 1 = “Yes”); and [3] “Training made me able to link clients successfully to HIV testing” (from 1 = “I strongly disagree” to 6 = “I strongly agree”).

We used the *Interprofessional Collaboration Scale (ICS)* to measure providers’ opinions and behaviors about IPC across service agencies (from “Strongly agree” to “Strongly disagree”) (Cronbach alpha = 0.84)*.* The ICS includes 49-items across five domains: interdependence, professional activities, flexibility, ownership of outcomes, and reflection on process [42]. Example statements include: “My colleagues refer clients to my agency,” “New programs arise from collaboration,” and “My colleagues and I often discuss strategies to improve our working relationships.” This study used the overall total score in the analysis where higher scores indicate higher interprofessional collaboration.

This study includes two variable levels: Level 1 consists of provider data and level 2 consists of agency data. Therefore, a two-level model was built using the following phases: First, the unconditional model (no independent variables) provided information about variability in the outcome between agencies. Next, provider level variables were included as fixed effects in a level one only model. Then, the agency level variables were added including level one and two fixed effects.

### Analysis

Raw data were imported from DatStat into IBM SPSS Statistics 24, and descriptive analyses were conducted to examine potential data errors, missing patterns, and data distribution. Two data-entry errors were identified and set to missing data. Missing data was less than 10%. All continuous variables met the collinearity assumption. There were ten outliers (studentized deleted residuals greater than ±2 standard deviations) which were removed from the analysis resulting in a final sample of 142 service providers across 13 community-based organizations.

Multilevel binary logistic regression analysis was conducted to assess the potential effects of IPC on links to HIV testing while simultaneously considering agency and provider demographics, self-efficacy, and training on HIV prevention and ARTAS. Multilevel modeling was used because providers are nested in agencies and thus not independent from agency contexts. Using “traditional” logistic regression is most likely to violate the independence of observations assumption, which may inflate the type I error rate. Multilevel modeling can manage such a nested data structure and provide appropriate standard errors [43]. The model fit of nested models was compared to determine which model had the best fit for the data.

## Results

### Agency characteristics

The total sample included 142 providers working in 13 agencies. Interviews with one administrator in each agency revealed that all agencies were community-based, nonprofit, private organizations. SUD treatment was offered by 46%; 16% were religious organizations, and the remaining agencies offered a variety of HIV-prevention and HIV-treatment services. Services provided included primary medical care for people living with HIV, outreach (educational seminars, street outreach, condom distribution), community intervention (health fairs, needle exchanges, food pantries, advocacy), interpersonal interventions (support groups, counseling, case management), and HIV testing. Table 1 displays agency demographics.

**Table 1:** Agency level variables: Descriptive statistics (*N* = 13)

Variables	N (%)
Agency Annual Budget	
$100,000 to $499,999	2 (15.4)
$500,000 to $999,999	1 (7.7)
$1,000,000 to $4,999,999	4 (30.8)
$5,000,000 to $10,000,000	2 (15.4)
More than $10,000,000	4 (30.8)
Agency primary services	
Substance Use Disorder treatment	8 (61.5)
HIV treatment and prevention	5 (38.5)

### Provider characteristics

The majority of the providers had been employed at their agencies for fewer than 5 years (59%), had received formal HIV-prevention training (59%), and earned between $25,000 and $49,999 annually. Overall, providers believed that formal training made them able to link clients successfully to HIV testing (mean = 3.8, standard deviation = 1.05). Providers also tended to agree that evidence-based HIV interventions can teach clients how to avoid HIV transmission (mean = 3.85, standard deviation = .83). Table 2 displays the service providers’ demographics. A total of 33% of participants reported linking clients to HIV testing in the previous 6 months. Of those reporting at least one linkage per week, as many as 41% reported making linkages several times a week, 21% once per week, and 38% once per month. Most providers reported linking more than 10 clients to HIV testing in the past 6 months (60%). Table 2 displays level 2 variables descriptive information.

**Table 2:** Service provider level variables: Descriptive statistics (*N* = 142)

Variables N(%)/ M (SD)	*Providers with no referrals*	*Providers with at least 1 referral*	Total
Age*	48.61 (10.79)	44.26 (12.40)	45.0 (12.2)
Gender			
Female	19 (59.6)	44 (53.7)	72 (54.5)
Male	28 (40.4)	51 (46.3)	60 (45.5)
Ethnicity			
Not Hispanic or Latino	37 (78.7)	65 (68.4)	96 (72.7)
Hispanic or Latino	10 (21.3)	30 (31.6)	36 (27.3)
Race			
White	33 (70.2)	67 (70.5)	38 (28.8)
Non-White	14 (29.8)	28 (29.5)	94 (71.2)
Highest Level of Education*			
High School Diploma / GED	22 (46.8)	32 (33.7)	48 (36.4)
Associate’s Degree	08 (17.0)	15 (15.8)	20 (15.2)
Bachelor’s Degree	05 (13.9)	31 (32.6)	35 (26.5)
Master’s Degree	11 (39.3)	17 (17.9)	28 (21.2)
Doctoral Degree	47 (100.0)	00 (00.0)	1 (0.8)
Income			
Less than $10,000	03 (06.4)	02 (02.1)	5 (3.8)
$10,000 to $24,999	11 (23.4)	12 (12.6)	20 (15.2)
$25,000 to $49,999	23 (48.9)	62 (65.3)	78 (59.9)
$50,000 to $74,999	10 (21.3)	14 (14.7)	24 (18.2)
$75,000 to $100,000	00 (00.0)	03 (03.2)	3 (2.3)
More than $100,000	00 (00.0)	02 (02.1)	2 (1.5)
Had HIV prevention training	27 (57.4)	57 (60.0)	79 (40.2)
Had ARTAS delivery training*	09 (19.1)	21 (22.10	29 (22.0)
Training enabled me to refer clients*			
I strongly disagree	01 (02.1)	04 (04.2)	04 (03.0)
I disagree	01 (02.1)	02 (02.1)	03 (02.3)
I tend to disagree	04 (08.5)	05 (05.3)	09 (06.8)
I tend to agree	17 (36.2)	12 (12.6)	24 (18.2)
I agree	11 (23.4)	36 (37.9)	47 (35.6)
I strongly agree	13 (27.70	36 (37.9)	45 (34.1)
HIV testing referral frequency			
Have not referred	47 (100.0)	00 (00.0)	47 (100.0)
Referred about once per month	00 (00.0)	36 (37.9)	36 (37.9)
Referred about once per week	00 (00.0)	20 (21.1)	20 (21.1)
Referred several times per week	00 (00.0)	39 (41.1)	39 (41.1)
Number of clients referred in past 6 months			
Less than 10 clients	47 (100.0)	38 (40.0)	85 (60.0)
10 to 20 clients	00 (00.0)	12 (12.6)	12 (08.4)
More than 20 clients	00 (00.0)	45 (47.4)	45 (31.6)
Interprofessional Collaboration Scale Total Score	95.61 (48.61)	101.81 (44.26)	100 (11.7)

*Statistically significant with *p* < .0

### Multilevel binary logistic regression analysis

A series of models were developed to examine if the addition of IPC would improve the likelihood of linkage to HIV testing over and above what might be expected from provider training and key demographics. Table 3 displays the results from the analysis. The addition of provider and agency demographics as well as training and self-efficacy had no significant contribution to the likelihood of providers making linkages to HIV testing (see models 1 to 3). However, the addition IPC scores showed a significant increase of model fit (model 3 to model 4) by 8.4%, F (12,119) = 1.821; *p* = .052. Altogether, the full model (model 4) showed a significantly better fit than the null model (i.e., no predictor model) by 11.4% with an overall model sensitivity of 90.2%. Providers in smaller agencies and agencies offering SUD services, and providers who were younger and who identified as White were more likely to make linkages to HIV testing. Those providers who received HIV training and who also perceived training helped prepare them effectively to make referrals were significantly more likely to refer clients to HIV testing. Finally, those who scored higher on the IPC scale reported significantly higher rates of links to HIV testing.

**Table 3:** Multilevel Logistic Regression models

*Fixed Effect*	Model 1		Model 2		Model 3		Model 4	
	B	OR (95% CI)	B	OR (95% CI)	B	OR (95% CI)	B	OR (95% CI)
Agency Income	−.652	.521 (.250–1.084)	−.804	.447 (.219–.916)	−1.150	.317 (.134–.749)	−1.922	.146 (.037–.572)
Agency Capacity	.610	1.841 (.316–10.725)	.648	1.911 (.349–10.463)	.714	2.042 (.288–14.498)	2.363	10.625 (.667–169.152)
Provider Age			−.039	.962 (.921–1.004)	−.045	.956 (.913–1.002)	−.131	.878 (.808–.953)
Provider Education			−.139	.870 (.554–1.367)	−.063	.939 (.560–1.576)	.231	1.260 (.638–2.491)
Provider Ethnicity			.340	1.405 (.351–5.621)	.032	1.032 (.229–4.659)	−.613	.542 (.065–4.481)
Provider Race			.444	1.559 (.455–5.346)	1.588	4.892 (.980–24.412)	3.584	36.000 (2.538–510.606)
Provider Gender			−.026	.974 (.365–2.602)	.259	1.295 (.438–3.825)	.540	1.716 (.394–7.480)
Provider Income			.693	2.000 (.974–4.104	.948	2.580 (1.151–5.783)	.720	2.055 (.763–5.533)
Self-Efficacy About Training					.740	2.096 (1.270–3.460)	1.063	2.895 (1.308–6.405)
Formal ARTAS Training					.410	1.507 (.370–6.134)	1.532	4.625 (.627–34.123)
Formal HIV Training					−1.015	.362 (.107–1.230)	−2.657	.070 (.011–.430)
IPC Scale							.234	1.263 (1.128–1.415)

*B* coefficient, *OR* odds ratio, *CI* confidence interval

## Discussion

This study sheds light on the influence of IPC on providers’ linkages to HIV testing above and beyond other provider- and agency-level factors. It also sheds light on the role of HIV training on linkage behavior. The findings, which focus on community-based organizations and providers in Newark, NJ, reflects similar findings from a larger study using data from 285 providers in 34 agencies in the urban environment of New York City. In the larger study, the results also show an increase in HIV testing overtime [44]. Below we offer a brief discussion and key implications concerning the sample characteristics, followed by the statistical model that we used.

### Sample characteristics influences on provider behavior

The study sample was diverse and reflected the population in Newark, with a majority of providers self-identifying as Black. The majority reported having at least an associate’s degree (62%). More than half the sample reported being 40 to 60 years of age, and there was a rough balance in gender, with 56% reporting being female. In the multiple hierarchical regression analysis, only age was a significant predictor of links to HIV services. Specifically, younger providers were more likely to link clients to such services. Perhaps this reflects changes in recent provider training to emphasize the importance of links to HIV testing. Nevertheless, it is critical that the workforce be trained to engage in IPC and to offer links to services on the care continuum in urban settings that contain a disproportionately high number of people living with HIV. We also found that providers who identified as White were more likely to make linkages to HIV testing. We will refrain from drawing conclusive implications from this finding, except to recommend that training to improve linkages to HIV testing need to be offered to providers of all race/ethnic identities.

A total of 33% of the service providers in this sample reported not making links to HIV testing in the previous 6 months. Understanding barriers to HIV testing referrals among these providers may shed light in ways to focus training and IPC interventions. For example, research suggests that when providers endorse interdependence among other providers in the same service system (e.g., mental health), establishment of more referrals and consultations are observed [45]. Nonetheless, research also shows that fear of losing clients to competitor agencies may inhibit providers from making HIV testing referrals [21]. Given that providers seek to improve the health of their clients and be effective in doing their jobs, embracing the concept of IPC, and continuing to invest time in training on evidence-based HIV interventions is recommended.

### Multilevel binary logistic regression

Multilevel binary logistic regression analysis supported our hypothesis that IPC is significantly associated with a higher likelihood of providers linking clients to HIV testing. Previous research has demonstrated the benefits of IPC and staff training on the delivery of services among healthcare providers [46–49]. However, no research has examined the direct association of IPC, as measured by a reliable scale, on linkages to HIV testing. The goal of this study was to examine whether IPC was significantly associated with links to HIV testing among providers of substance-use treatment and HIV-prevention services, after controlling for the effects of demographics and provider training, attitudes, and self-efficacy. Previous research shows that increased treatment adherence and HIV testing are the most effective interventions to reduce HIV incidence [11]; therefore, it follows that community-based organizations ought to maximize IPC so as to improve HIV testing rates in urban cities similar to Newark, NJ.

The analysis showed that the addition of provider training in the specific areas tested as a factor only improved the prediction model by 3%, after controlling for agency and provider demographics. However, the literature suggests that, in general, on-the-job training has the potential to substantially increase the efficacy of links by reducing cost and by making links more specific and flexible [50, 51]. We submit that our findings may reflect trends that are based on environment variables (e.g., political trends, funding streams, culture) which have not been tested by the current model. As evidence, we report that using the same measurements in a sample of providers in New York City; in multivariate analysis, after adjusting for IPC, linkage training was associated with more frequent services [52]. Further research using longitudinal designs and a variety of measurement strategies is needed to confirm these findings and more thoroughly examine the role of provider efficacy and formal training in Newark, NJ, and beyond.

### Limitations and strengths

This study used cross-sectional data collected within single geographic location (in Newark NJ); therefore, we were unable to establish neither causality nor generalizability to other regions or cities. However, the data suggest that training providers to develop knowledge about and more-positive attitudes toward IPC may help improve the overall frequency of links. It is important to note that the outcome variables were self-reported and many variables had a wide range of confidence intervals due to a relatively small sample size. The dichotomous outcome variable does not provide information about the variation on number of linkages among providers who reported making at least one referral. Therefore, study’s findings should be interpreted with caution. Future research needs include larger samples and focus on teasing out specific domains of IPC that may encourage different types of providers to link their clients to HIV testing. Given existing research supporting the association between early HIV detection (i.e., testing), engagement in HIV care, and viral suppression, [8, 53] it is important to increase our understanding of IPC as a predictor of links to HIV testing, particularly in urban settings and agencies focusing on marginalized clients facing SUD.

In spite of stated limitations, this study includes several strengths. The study drew a sample of providers from 13 different organizations which allowed us to reflect collaboration across agencies that provide HIV and substance use disorder treatment. The study used CBPR principles and best practices by engaging two community collaborative boards that offered the perspectives of health service providers and consumers in every step of the study. The study used a computer assisted data collection strategy in in-person interviews to maximize data confidentiality, validity, and reliability. Hence, findings from these data offer a significant contribution to the field of HIV prevention and treatment.

## Conclusions

Identifying individuals living with HIV residing in marginalized communities that are disproportionately affected by high HIV-infection rates is imperative to achieve health and social justice in these communities. Our findings and the literature indicate that interprofessional collaboration may be a significant predictor of increased links to HIV testing, which in turn is a predictor of improved health and HIV-prevention outcomes. More research is needed to identify ways to promote IPC in marginalized communities.

## Supplementary Information

**Additional file 1.** Study Surveys.

## Data Availability

The datasets used and/or analyzed during the current study are available from the corresponding author on reasonable request.

## Abbreviations

ARTAS: Antiretroviral Treatment Access Studies
IPC: Interprofessional-collaboration
HIV: Human Immunodeficiency Virus
SUD: Substance-Use Disorder
AIDS: Acquired Immunodeficiency Syndrome
ART: Antiretroviral Therapies
CBPR: Community-Based Participatory Research
NCCB: Newark Community Collaborative Board
ICCB: Implementation Community Collaboration Board
ICS: Interprofessional Collaboration Scale
