# Supplementary material for: Interprofessional collaboration associated with frequency of life-saving links to HIV continuum of care services in the urban environment of Newark, New Jersey
Source: BMC Health Serv Res. 2020 Nov 7;20:1014. doi: 10.1186/s12913-020-05866-3 (PMC7648428; doi:10.1186/s12913-020-05866-3)
Supplement: Supplementary file 1 — Additional file 1. Study Surveys. [file 12913_2020_5866_MOESM1_ESM.zip › Project ICI NJ Administrator SurveyR4.pdf]

PROJECT ICI – INTERAGENCY COLLABORATION for IMPLEMENTATION

ADMINISTRATOR SURVEY

KEY for all Likert scale questions
(Except for existing scales)

1 = Strongly Agree
2 = Agree
3 = Tend to Agree
4 = Tend to Disagree
5 = Disagree
6 = Strongly Disagree

Organization size and capacity

1. What category best describes the type of agency/organization at which you are currently employed: (Check one)

- Private, nonprofit organization
- Private, for-profit organization
- Unit of state government
- Unit of local county or municipal government
- Unit of tribal government
- Federal Department of Veteran Affairs
- Other federal agency
- Other public cooperation

2. What categories best describe your primary agency setting: (Check all that apply)

- Religious
- Alcohol and Drug Abuse Treatment or Prevention Program
- Family/Children’s Service Agency
- Social Service Agency
- Hospital
- Mental System/Community Mental Health Clinic
- Community Health Clinic (primary care/urgent care)
- AIDS service organization
- Housing Program
- Other (please specify)

3. On average, what category best describes your agency’s annual budget:

- $50,000 to $ 99,999
- $100,000 to $499,999
- $500,000 to $999,999
- $1 million to $5 million
- $5 million to $10 million
- More than $10 million

4. In what geographic area does your agency provide HIV prevention programs/services? (Check all that apply)

- Essex County
- Union County
- City of Newark
- State -wide
- other

5. How many employees provide direct clinical or case management services to clients?

- Fewer than 25 staff members
- 26 to 50 staff members
- 50 to 100 staff members
- More than 100 staff members

6. In what languages are services provided at your agency? (Check all that apply)
- English
- Spanish
- French
- Arabic
- Mandarin or Cantonese
- French-based Creole
- Other

7. What type of populations does your agency target for HIV prevention (Check all that apply)
- Youth (ages 13-24)
- Aged (age 60 and over)
- Immigrants
- Substance users
- Individuals involved in the criminal justice system
- Homeless persons
- Sex workers
- Women
- Men
- Adults with mental illness
- People affected by domestic violence
- Transgender persons
- Gay Men
- HIV Seropositives
- Other

8. In your estimation, clients receiving HIV services from your agency comprise:
- Less than 10% of your agency's total client population
- 10% to 25% of your agency's total client population
- 26% to 50% of your agency's total client population
- 51% to 75% of your agency's total client population
- More than 75% of your agency's total client population

9. Does your agency currently provide child care for clients? Y/N

10. Does your agency currently offer evening hours for clients? Y/N

11. Does your agency currently carry liability/insurance coverage? Y/N

12. Lack of funding currently affects my agency's ability to add new services/programs.
(1 = Strongly Agree; 2 = Agree; 3 = Tend to Agree; 4 = Tend to Disagree; 5 = Disagree; 6 = Strongly Disagree)

13. Lack of funding currently affects my agency's ability to expand existing services/programs.
(1 = Strongly Agree; 2 = Agree; 3 = Tend to Agree; 4 = Tend to Disagree; 5 = Disagree; 6 = Strongly Disagree)

14. What type(s) of HIV-related services does your agency provide? (Check all that apply)

<u>Primary medical care for HIV positives</u>

<u>Outreach</u>

- Educational seminars
- Condom distribution
- Venue-based
- Street outreach

<u>Community Intervention</u>

- Health fairs
- Needle exchange
- Food pantry
- Advocacy

<u>Interpersonal Interventions</u>

- Support groups
- Family counseling
- Couples counseling
- Individual counseling
- Case management

Staff preparedness

15. Does your agency provide staff the following opportunities (mark all that apply)

- Formal mentoring
- Individual supervision
- Group supervision
- Computer-based/Web-based trainings
- Seminars and workshops on HIV prevention
- Seminars and workshops on substance abuse
- Seminars and workshops on sexual diversity issues and competency
- Seminars and workshops on racial/ethnic diversity issues and competency

Research experience

16. Has your agency been involved in HIV-related program evaluation? (If yes, complete questions below. If no, go to #19.) Yes _____ No _____ How long ago?_____

17. In how many HIV-related program evaluations has your agency been involved in the past three years? ____ evaluations

18. Over the past three years, what was the average percentage of staff involved in HIV-related program evaluation?

- None
- 10% to 25% of staff
- 26% to 50% of staff
- 51% to 75% of staff
- More than 75% of staff

19. Has your agency been involved in HIV-related research projects connected to a university or research institute?
Yes______ No________

20. Has your agency been involved in HIV-related research projects connected to another, non-academic research-related organization?
Yes _____ No _____

21. In the past three years, in how many HIV-related research projects has your agency been involved?

22. In the past three years, what was the average percentage of staff involved in HIV-related research projects?

- None
- 10% to 25% of staff
- 26% to 50% of staff
- 51% to 75% of staff
- More than 75% of staff

23. Do your agency's program evaluation and/or research projects typically involve outside researchers?
Yes ____ No _____

24. Does your agency have internal researchers?
Yes ____ No _____

EBIs

25. Does your agency offer Effective Behavioral Intervention (EBIs) funded by the Centers for Disease Control and Prevention (CDC) or the State of New Jersey?
Yes ____ No _____

. Which ones? Only answer if checked “yes” for question 25)

- CLEAR
- CONNECT
- d-up
- Healthy Relationships
- Mpowerment
- Partnership for Health
- PCC
- POL
- PROMISE
- RAPP
- RESPECT
- SHIELD
- START
- VOICES/VOCES
- WILLOW
- 3MV
- I do not know
- My agency doesn’t offer DEBIs

26. Your agency is successful at matching client needs with EBIs.
(1 = Strongly Agree; 2 = Agree; 3 = Tend to Agree; 4 = Tend to Disagree; 5 = Disagree; 6 = Strongly Disagree)

27. Your agency is successful at matching client demographics with EBIs.
(1 = Strongly Agree; 2 = Agree; 3 = Tend to Agree; 4 = Tend to Disagree; 5 = Disagree; 6 = Strongly Disagree)

28. Your agency measures client outcomes within its HIV programs by assessing: (mark all that applies)

Retention____
Attendance____
Changes in mental health___
Changes in physical health____
Changes in sexual risk behaviors____
Changes in substance/alcohol use_____
Changes in housing_____
Changes in employment______
Please explain how you assess the above outcomes ____

29. Your agency utilizes client outcomes, such as those in the previous question, to guide program decisions. Yes / No
Please explain how ____

30. Your agency utilizes client satisfaction surveys to guide program decisions. Yes / No
Please explain how ____

31. Your agency develops programs based on research evidence. Yes / No

Please explain how ______________________________________

32. To the best of your knowledge, in the past 6 months, how many clients have been referred to your agency so that they could access an EBI that you offer? ________

33. Which agencies made the referrals?

34. To the best of your knowledge, in the past 6 months, how many clients has your agency referred to another agency so that they could access an EBI that that agency offers? ________

35. To which agencies were the referrals made?

African American Office of Gay Concerns	Newark Beth Israel Medical Center
Bergen Regional	Newark Community Health Center
Bridges	Newark Department of Child and Family Well-Being Homeless HealthCare Program
Broadway House (Newark AIDS Consortium)	Newark Emergency Services
Covenant House	North Jersey Aids Alliance (NJCRI)
Cura Inc Outpatient And Residential Short Term	Proceed Inc
East Orange Substance Abuse Treatment Program	Renaissance House Inc Youth And Family Treatment Center
Hyacinth Aids Foundation - Newark	Restoration Center
Integrity, Inc.	St. Michael's Medical Center-Peter Ho Clinic
Kintock Group	St. Bridget's Residence
La Casa Don Pedro	Tully House
Lennard Clinic	Turning Point
New Hope Baptist Church	Other
	None of the above

Staff Preparedness

36. Please indicate whether your agency provides its staff with any of the following opportunities. (Please check all that apply.)

Formal mentoring
Individual supervision
Group supervision
Computer-based/Web-based trainings
Seminars and workshops on HIV prevention
Seminars and workshops on substance abuse
Seminars and workshops on sexual diversity issues and competency
Seminars and workshops on racial/ethnic issues and competency

37. Please tell us how often your agency provides staff with any of the following opportunities:

We provide 1 seminar per month
We provide 2 seminars per year
We provide 1 seminar per year
We provide 1 seminar every other year

ARTAS

Please read the definition of ARTAS below and answer the questions that follow.

ARTAS refers to Anti-Retroviral Treatment and Access to Services, an individual level, multi-session, time-limited intervention to link individuals who have been recently diagnosed with HIV to medical care.

36. Does your agency help clients access HIV testing and primary care using the ARTAS model (Anti-Retroviral Treatment and Access to Services)? (if not go to question 42)

No
Yes
I don’t know
Other Funding Source ____________________________

37. When did your agency start linking clients to HIV testing and primary care using the ARTAS model ?

More than 2 years ago
1 to 2 years ago
6 months to 1 year ago
Within the past 6 months

38. Who provided the funding to train your staff in the ARTAS model?

CDC
Department of Health
Another funder source (please specify) _____________________

39. Does your agency have a tracking system to determine how many of your clients accessed HIV testing and/or primary care (ARTAS)?

Yes
No

40. Does your agency link clients to HIV testing and primary care?

More than 2 years ago
1 to 2 years ago
6 months to 1 year ago
Within the past 6 months

41. Does your agency have a tracking system to determine how many of your clients accessed HIV testing and/or primary care?

Yes
No

42. What is the tracking system?

Electronic chart record
Access Database
Excel/Word Document
Logs
Hand-written Progress Notes
Other (please specify)____________________________

44. In general, how do providers in your agency make referrals? (Check all that apply)

My staff hand the client a card with contact information.
My staff make a call/send an email to schedule an appointment for the client after the client leaves my office.
My staff make a call/send an email to schedule an appointment for the client while the client is still in my office.
My staff ask the client to make a call or send an email to schedule the appointment in my presence.
My staff offer the client information (brochures/pamphlets/Web site).
My staff escort the client to another service provider/program/agency.
My staff do not refer clients
