# Supplementary material for: Interprofessional collaboration associated with frequency of life-saving links to HIV continuum of care services in the urban environment of Newark, New Jersey
Source: BMC Health Serv Res. 2020 Nov 7;20:1014. doi: 10.1186/s12913-020-05866-3 (PMC7648428; doi:10.1186/s12913-020-05866-3)
Supplement: Supplementary file 1 — Additional file 1. Study Surveys. [file 12913_2020_5866_MOESM1_ESM.zip › Project ICI_NJ Provider_SurveyR4.pdf]

New Jersey Project ICI - Baseline Survey

1. Agency Identification Number:_____

Organization size and capacity

2. Please give the name of your agency:____________

3. Which of these categories best describes the type of agency where you work? (Please check one.)

Private, nonprofit organization
Private, for-profit organization
Unit of state government
Unit of local county or municipal government
Unit of tribal government
Federal Department of Veteran Affairs
Other federal agency
Other public cooperation

4. Which categories best describe your agency setting? (Please check all that apply.)

Religious
Alcohol and Drug Abuse Treatment or Prevention Program
Family/Children's Service Agency
Social Service Agency
Hospital
Mental System/Community Mental Health Clinic
Community Health Clinic (primary care/urgent care)
AIDS service organization
Housing Program
Other (please specify)

5. Which category best describes your agency's annual budget?

$50,000 to $ 99,999
$100,000 to $499,999
$500,000 to $999,999
$1 million to $5 million
$5 million to $10 million
More than $10 million

6. In what geographic area does your agency provide HIV prevention programs/services? (Check all that apply)

Essex County
Union County
City of Newark
State-wide
Other

7. How many employees provide direct clinical or case management services to clients?

Fewer than 25 staff members
26 to 50 staff members
50 to 100 staff members
More than 100 staff members

8. In what languages are services provided at your agency? (Check all that apply)

English
Spanish
French
Arabic
Mandarin or Cantonese
French-based Creole
Other

9. What type of populations does your agency target for HIV prevention (Check all that apply)

Youth (ages 13-24)
Aged (age 60 and over)
Immigrants
Substance users
Individuals involved in the criminal justice system
Homeless persons
Sex workers
Women
Men
Adults with mental illness
People affected by domestic violence
Transgender persons
Gay Men
HIV Seropositives
Other

10. What percentage of your clients receives HIV services from your agency?

Less than 10% of your agency's total client population
10% to 25% of your agency's total client population
26% to 50% of your agency's total client population
51% to 75% of your agency's total client population
More than 75% of your agency's total client population

11. Does your agency currently provide child care for clients?

Yes
No

12. Does your agency currently offer evening hours for clients?

Yes
No

13. Does your agency currently carry liability/insurance coverage?

Yes
No

14. Lack of funding currently affects my agency's ability to add new services/programs.
(1 = Strongly Agree; 2 = Agree; 3 = Tend to Agree; 4 = Tend to Disagree; 5 = Disagree; 6 = Strongly Disagree)

15. Lack of funding currently affects my agency's ability to expand existing services/programs.
(1 = Strongly Agree; 2 = Agree; 3 = Tend to Agree; 4 = Tend to Disagree; 5 = Disagree; 6 = Strongly Disagree)

16. What type(s) of HIV-related services does your agency provide? (Check all that apply)

<u>Primary medical care for HIV positives</u>

<u>Outreach</u>

Educational seminars
Condom distribution
Venue-based
Street outreach

<u>Community Intervention</u>

Health fairs
Needle exchange
Food pantry
Advocacy

<u>Interpersonal Interventions</u>

Support groups
Family counseling
Couples counseling
Individual counseling
Case management

Research experience

17. In the past three years, in how many research projects and/or program evaluations has your agency been involved (e.g., cross sectional, clinical trial, qualitative evaluation, pre- and post-tests)? Please count any project in which your agency currently may be involved.

More than 20 research projects
16-20 research projects
11-15 research projects
5-10 research projects
Less than 5 research projects
My agency has not been involved in a research project yet

18. In the past three years, what was the average percentage of staff involved in HIV-related program evaluation?

None
10% to 25% of staff
26% to 50% of staff
51% to 75% of staff
More than 75% of staff

19. Do your agency's research projects and/or program evaluations typically involve outside researchers?

Yes
No

20. Do your agency's research projects and/or program evaluations typically involve outside researchers?

Yes
No

21. Does your agency have internal researchers?

Yes
No

22. Has your agency been involved in HIV-related research projects connected to a university or research institute?

Yes
No

23. Has your agency been involved in HIV-related research projects connected to another, non-academic research-related organization?

Yes
No

EBIs

To begin this section, please review the definition of DEBIs below.

The Centers for Disease Control and Prevention (CDC) has identified behavioral interventions that are effective in helping individuals avoid HIV infection.They are called DEBIs. DEBIs are delivered using manuals that tell you exactly what to do in sessions with individuals or groups of clients in community agencies.

24. Does your agency offer any of the following DEBIs funded by the Centers for Disease Control and Prevention (CDC) or the State of New Jersey? (Check all that apply)

CLEAR
CONNECT
D-up
Healthy Relationships
Mpowerment
Partnership for Health
PCC
POL
PROMISE
RAPP
RESPECT
SHIELD
START
VOICES/VOCES
WILLOW
3MV
I do not know
My agency doesn’t offer DEBIs

25. Your agency is successful at matching client needs with **CLEAR**.
(1 = Strongly Agree; 2 = Agree; 3 = Tend to Agree; 4 = Tend to Disagree; 5 = Disagree; 6 = Strongly Disagree)

26. Your agency is successful at matching client demographics with **CLEAR**.
(1 = Strongly Agree; 2 = Agree; 3 = Tend to Agree; 4 = Tend to Disagree; 5 = Disagree; 6 = Strongly Disagree)

Answer 24 & 25 for each of the DEBIS at your agency.

27. Your agency measures client outcomes within its HIV programs by assessing: (mark all that applies)

Retention____
Attendance____
Changes in mental health___
Changes in physical health____
Changes in sexual risk behaviors____
Changes in substance/alcohol use_____
Changes in housing_____
Changes in employment______
Please explain how you assess the above outcomes _____________________________________

28. Does your agency have a tracking system to measure client outcomes?

Yes
No

29. What is the tracking system?

Electronic Chart Record
Access Database
Excel/Word document
Logs
Hand-written Progress Notes
Other (please specify)

30. Does your agency use information about client outcomes to guide program decisions?

Yes (please specify how)
No

31. Does your agency use client satisfaction surveys to guide program decisions?

Yes (please specify how)
No

32. In general, does your agency develop programs based on research evidence?

Yes (please specify how)
No

33. With which of these agencies do you have formal or informal agreements about referral exchange? (Please check all that apply. Please note that there is a "None of the above" option.)

African American Office of Gay Concerns	Newark Beth Israel Medical Center
Bergen Regional	Newark Community Health Center
Bridges	Newark Department of Child and Family Well-Being Homeless HealthCare Program
Broadway House (Newark AIDS Consortium)	Newark Emergency Services
Covenant House	North Jersey Aids Alliance (NJCRI)
Cura Inc Outpatient And Residential Short Term	Proceed Inc
East Orange Substance Abuse Treatment Program	Renaissance House Inc Youth And Family Treatment Center
Hyacinth Aids Foundation - Newark	Restoration Center
Integrity, Inc.	St. Michael's Medical Center-Peter Ho Clinic
Kintock Group	St. Bridget's Residence
La Casa Don Pedro	Tully House
Lennard Clinic	Turning Point
New Hope Baptist Church	Other
	None of the above

Staff Preparedness

34. Please indicate whether your agency provides its staff with any of the following opportunities. (Please check all that apply.)

Formal mentoring
Individual supervision
Group supervision
Computer-based/Web-based trainings

Seminars and workshops on HIV prevention
Seminars and workshops on substance abuse
Seminars and workshops on sexual diversity issues and competency
Seminars and workshops on racial/ethnic issues and competency

35. Please tell us how often your agency provides staff with any of the following opportunities:

We provide 1 seminar per month
We provide 2 seminars per year
We provide 1 seminar per year
We provide 1 seminar every other year

ARTAS

Please read the definition of ARTAS below and answer the questions that follow.

ARTAS refers to Anti-Retroviral Treatment and Access to Services, an individual level, multi-session, time-limited intervention to link individuals who have been recently diagnosed with HIV to medical care.

36. Does your agency help clients access HIV testing and primary care using the ARTAS model (Anti-Retroviral Treatment and Access to Services)? (if not go to question 42)

No
Yes
I don't know
Other Funding Source ____________________________

37. When did your agency start linking clients to HIV testing and primary care using the ARTAS model ?

More than 2 years ago
1 to 2 years ago
6 months to 1 year ago
Within the past 6 months

38. Who provided the funding to train your staff in the ARTAS model?

CDC
Department of Health
Another funder source (please specify) _____________________

39. Does your agency have a tracking system to determine how many of your clients accessed HIV testing and/or primary care (ARTAS)?

Yes
No

40. Does your agency link clients to HIV testing and primary care?

More than 2 years ago
1 to 2 years ago
6 months to 1 year ago
Within the past 6 months

41. Does your agency have a tracking system to determine how many of your clients accessed HIV testing and/or primary care?

Yes
No

42. What is the tracking system?

Electronic chart record
Access Database
Excel/Word Document
Logs
Hand-written Progress Notes
Other (please specify)____________________________

43. In general, how do providers in your agency make referrals? (Check all that apply)

My staff hand the client a card with contact information.
My staff make a call/send an email to schedule an appointment for the client after the client leaves my office.
My staff make a call/send an email to schedule an appointment for the client while the client is still in my office.
My staff ask the client to make a call or send an email to schedule the appointment in my presence.
My staff offer the client information (brochures/pamphlets/Web site).
My staff escort the client to another service provider/program/agency.
My staff do not refer clients

You have completed the survey. Please click on the orange "submit" button below.
